# A giant virus in a small lake: characterization of a new tropical algal virus expands the geographical and genetic diversity of the Brazilian virosphere

**DOI:** 10.1128/jvi.00318-26

**Published:** 2026-05-28

**Authors:** Clécio Alonso da Costa Filho, Isadora Luiza de Jesus Canto, Bruna Barbosa Fagundes Botelho, Lethícia Ribeiro Henriques, João Victor Rodrigues Pessoa Carvalho, Ellen Gonçalves de Oliveira, Sara Arêdes da Cunha Souza, Júlia Winkler Souza, Mauricio Teixeira Lima, Felipe Campos de Melo Iani, Amanda Haisi, João Pessoa Araújo Júnior, Rodrigo Araújo Lima Rodrigues

**Affiliations:** 1 Laboratório de Vírus, Departamento de Microbiologia, Instituto de Ciências Biológicas, Universidade Federal de Minas Gerais, Belo Horizonte, MG, Brazil; 2 Núcleo de Apoio Técnico ao Ensino, Pesquisa e Extensão, Químicas e Farmacêuticas, Instituto de Ciências Ambientais, Universidade Federal de São Paulo, Diadema, SP, Brazil; 3 Serviço de Virologia e Riquetsioses, Fundação Ezequiel Dias 282795, Belo Horizonte, MG, Brazil; 4 Instituto de Biotecnologia, Universidade Estadual Paulista Júlio de Mesquita Filho 28108, Botucatu, SP, Brazil; Michigan State University, East Lansing, Michigan, USA

**Keywords:** *Alphachlorovirus*, giant virus, algae virus, aquatic virus, biodiversity

## Abstract

Chloroviruses are large double-stranded DNA (dsDNA) viruses that infect *Chlorella*-like green algae in freshwater environments. Despite being extensively studied in the Northern Hemisphere, little is known about their diversity and biology in tropical regions. In this study, we report the isolation and characterization of a novel chlorovirus, named Chlorovirus BR-MG-N01, infecting *Chlorella variabilis* NC64A. The virus was recovered from a freshwater lake in Guanhães, Minas Gerais, Brazil, located in a transition zone between the Atlantic Forest and Cerrado biomes. Electron microscopy revealed icosahedral particles of approximately 160 nm in diameter. The viral growth profile showed a 2-log increase after 24 h of infection. BR-MG-N01 demonstrated high thermal stability, remaining viable from −80°C to 56°C, but showed low tolerance to ultraviolet radiation, with a rapid decrease in titer after 3 min of exposure. Genome sequencing revealed a 318 kbp dsDNA genome containing 384 coding sequences. Gene annotation identified a high number of endonucleases, two of which are putatively novel, as well as genes related to transcription and oxidative stress resistance. Phylogenetic analysis based on the DNA polymerase gene placed BR-MG-N01 within the *Alphachlorovirus* subgenus, closely related to the species *Chlorovirus vanettense*. Comparative genomic analysis revealed strong synteny and high sequence similarity with other members of this species. The isolation of Chlorovirus BR-MG-N01 expands the known distribution of chloroviruses and highlights the need for further exploration of tropical environments to uncover the ecological and evolutionary diversity of these enigmatic aquatic viruses.

**IMPORTANCE:**

Chloroviruses are large dsDNA viruses that infect algae, with limited available research in tropical environments. This work helps address that gap by presenting the first detailed genomic and biological characterization of a native chlorovirus from Brazil. We show that Brazilian environments harbor viral lineages as complex and diverse as those described in well-studied regions of the Northern Hemisphere. By documenting the isolate’s morphology, stability, replication profile, genomic architecture, and evolutionary placement, this study establishes the basis for future ecological and evolutionary research on algal viruses in the country. Overall, the discovery highlights a hidden viral diversity in Brazilian ecosystems and reinforces the importance of investigating microorganisms from tropical and understudied habitats.

## INTRODUCTION

Chloroviruses are large, plaque-forming, double-stranded DNA (dsDNA) viruses that infect *Chlorella*-like green algae in freshwater environments. They exhibit an icosahedral capsid with a spike-like structure in one of the vertices that mediates host interaction (1). These viruses have a linear genome ranging from ~280 to 410 kbp, encoding between 300 and 416 proteins (2). Nearly 60% of the genes identified in chloroviruses have no known homologs in other organisms, and a large proportion of them also have unknown functions (~66%), highlighting a vast area for further investigation. This reflects the extensive genetic diversity characteristic of these viruses (3).

Viruses play a central role in the control of microbial communities (4). They are key regulators of algal bloom dynamics and represent mortality factors for bacteria that are as significant as protozoan predation. Moreover, through their influence on the food web, viruses affect global biogeochemical cycles (5, 6). Chloroviruses infect and lyse the cells of their hosts, contributing to algal mortality and influencing the dynamics of microbial communities. These viruses exhibit complex ecological traits and a unique lifestyle. Their hosts can occur as free-living organisms or as symbionts of other organisms, such as *Paramecium bursaria* and *Acanthocystis turfacea* (1). Once the algae are inside their holobiont, they are protected from viral infection; however, the protists can be predated (e.g., by a copepod) and catalyze viral infection (7–9). Chloroviruses particles attract *P. bursaria* through a still unknown mechanism, a process called virotaxis, which increases the possibility for viruses to attach to the protist membrane and subsequently finding an available algal host (10). In addition, chloroviruses are consumed by a variety of protists (e.g., *Halteria* sp.), serving as food in a complex microbial loop, a process called virovory (11).

The best studied chlorovirus, Paramecium bursaria Chlorella virus 1 (PBCV-1), was isolated in 1981 and recognized as the model virus of the genus *Chlorovirus* in the 1990s (12). Phylogenetic analyses indicate that chloroviruses diverge into three monophyletic clades corresponding to the host algal species they infect: *Chlorella variabilis* strains NC64A and Syngen 2-3 (alphachloroviruses), *Micractinium conductrix* Pbi (betachloroviruses), and *Chlorella heliozoae* SAG 3.83 (gammachloroviruses) (2, 13). The vast majority of the known chloroviruses have been isolated from water samples from the Northern Hemisphere, particularly from alkaline lakes in the Sandhills region in Nebraska, USA (2, 3, 14, 15). Genomic and evolutionary analyses comparing several isolates from this environment have shown that these viruses have unprecedented diversity, even within the same ecosystem (2, 14, 15). Only four of these genomes belong to chloroviruses isolated from inland waters in the Southern Hemisphere, and almost nothing is known about their biology. In Brazil, only one genome sequence of a chlorovirus isolated from a sample collected in São Paulo is publicly available, but nothing is known about the biology of this isolate, highlighting a major gap in knowledge about these viruses in the country (13, 15). Therefore, obtaining isolates from tropical regions such as Brazil, a country renowned for its biodiversity, is of great importance to better understand the extent of the genetic and biological diversity of these viruses. Furthermore, the isolation of new chloroviruses will help deepen our understanding of their role in aquatic ecosystems, since known viral strains exhibit substantial variations in ecological traits, evidencing that closely related viruses are biologically different and may not be representative of their relatives (16).

In this study, we isolated and characterized a novel chlorovirus infecting the microalgae *Chlorella variabilis* NC64A and named it as Chlorovirus BR-MG-N01. The virus was isolated from a lake in the municipality of Guanhães, Minas Gerais, Brazil. The lake is located in a peri-urban area, relatively distant from the city, within a transition zone between the Atlantic Forest and Cerrado biomes. After virus purification, we evaluated several aspects of its biology and genome, being the first in-depth characterization of a giant algal virus isolated in Brazil, providing insights into the still poorly understood diversity and complexity of these important aquatic viruses in the Southern Hemisphere.

## MATERIALS AND METHODS

### Environmental sample collection

The environmental water samples analyzed in this study were collected in the Brazilian state of Minas Gerais in 2023. Samples were collected from different aquatic environments, including lakes and rivers across multiple regions of the state, encompassing areas within the Atlantic Forest and Cerrado biomes. The viral isolate analyzed in this study was obtained from a collection of samples gathered from lakes in the peri-urban area of the city of Guanhães (18°48′50.5″S 42°57′17.1″W), a region influenced by the Doce River basin, one of Brazil’s most important river systems. Using Falcon tubes, 50 mL of water samples were collected at each point and stored at 4°C until further use in the Virus Laboratory of the Federal University of Minas Gerais (UFMG). This study is registered at the National System for the Management of Genetic Heritage and Associated Traditional Knowledge (SisGen) under the accession number A048F34.

### Host and virus acquisition

For viral prospecting, *Chlorella variabilis* NC64A was cultivated in Modified Bold’s Basal Medium (MBBM) with tetracycline (filter sterilized, 10 µg/mL final concentration) at 25°C, under continuous illumination. PBCV-1 was used as experiment control in viral assays. Purified chlorovirus and the *Chlorella* strain were kindly provided by Professor James L. Van Etten from the Nebraska Center for Virology, University of Nebraska–Lincoln, USA (17).

### Virus isolation and purification

The water samples were filtered through a 0.45-µm membrane, and 1 mL of each sample was used to infect cultures of *C. variabilis* in the exponential growth phase (~10⁸ cells/mL) using the double-layer method (17). Plates were incubated in continuous light at 25°C for up to 14 days to evaluate the formation of lysis plaques.

For viral isolation and titration, we used plaque assay, as described previously with modifications (13, 17). Briefly, a lysis plaque was collected from the algal lawn, transferred to a tube containing 0.5 mL of PBS solution, and purified through rounds of infection and plating until only one plaque morphology was observed. Viral titration was performed by inoculating serially diluted viral samples using the double-layer method in six-well plates.

Once the viral titer was determined, an infection at a multiplicity of infection (MOI) of 0.01 Plaque Forming Units (PFU) per cell was carried out in 4 × 10^10^ cells incubated under constant light at 25°C with agitation until the observation of cytopathic effect (clearing of algae culture). The resulting lysate was centrifuged at 4,000 × *g* for 10 min. Subsequently, 1 mL of 1% Triton X-100 was added in the cell pellet to promote cell lysis and the release of non-released particles and centrifuged under the same conditions. The resulting content was then combined with the previously collected supernatant and filtered through a 0.45-µm membrane, followed by ultracentrifugation at 24,000 × *g* for 60 min at 4°C (SW 32 Ti rotor, Beckman Coulter, Inc., USA). Viral particles were resuspended in 15 mL of PBS pH 7.2 and were further purified on a 10%–40% sucrose gradient (in distilled water) by centrifugation at 36,000 × *g* for 20 min using the same rotor as above (18). The purified viral band was collected and resuspended in PBS solution, and the concentration of purified viral particles (viral stock) was determined by titration, yielding a titer of 3 × 10⁹ PFU/mL.

### Viral stability and yield assays

To evaluate the stability profile of the isolated virus, biological assays were performed under different temperature conditions and UV radiation exposure. For the temperature assays, 100 µL of purified viral stock was aliquoted into microtubes and subjected to various temperatures for 10 min. Ultra-low freezers were used for the –80°C and –30°C conditions. Samples exposed to 4°C were stored in a standard refrigerator, while those exposed to 37°C, 56°C, and 100°C were placed in a thermoblock. After temperature exposure, the viral samples were serially diluted and titrated. For the UV radiation resistance tests, 100 µL of purified viral stock was placed in six-well plates and exposed to a UV light source (15 W, 15 cm from the lamp) inside a biological safety cabinet under room temperature. Viral contents were removed at defined time intervals: 1, 2, 3, 4, 5, 10, and 15 min of exposure, serially diluted and titrated.

To evaluate the viral yield after replication, infections at MOI = 10 were conducted using 500 µL of *Chlorella variabilis* NC64A culture (containing 1 × 10⁸ total cells) in microtubes. The tubes were maintained on an orbital shaker, and after 30 min of viral adsorption, samples were collected and centrifuged at 0 and 24 h post-infection (hpi). The supernatants were then harvested and titrated.

### Electron microscopy

The purified viral isolate was subjected to transmission and scanning electron microscopy to observe viral morphology and aspects of its replication cycle. For transmission electron microscopy (TEM), an infection at MOI = 1 PFU/cell in a test tube was kept under rotation (100 rpm) for 24 h. The infected culture was centrifuged at 4,000 × *g* for 10 min. The supernatant was discarded, and the pellet was resuspended in 1.5 mL of Karnovsky solution (2% paraformaldehyde and 2.5% glutaraldehyde in 0.1 M phosphate buffer, pH 7.3). After 4 h of fixation, the pellet was washed with 0.1 M phosphate buffer (pH 7.3). The sample was sent to the Microscopy Center of UFMG, where it was post-fixed with osmium tetroxide, embedded in resin, sectioned, and contrasted. Visualization was performed using a Tecnai G2-12-Spirit BioTwin FEI – 120 kV transmission electron microscope. Micrographs were analyzed, and particles were measured using ImageJ software (19). In addition, the isolate was analyzed by negative-staining electron microscopy. A purified virus sample was sent to the Microscopy Center, where it was deposited on a carbon-coated microscopy grid. The sample was then stained with uranyl acetate and visualized using the aforementioned Tecnai microscope.

For scanning electron microscope (SEM), 10 μL of purified viral sample with a total titer of 1 × 10^6^ PFU was applied to a round glass coverslip coated with poly-L-lysine and fixed with 2.5% glutaraldehyde in 0.1 M sodium phosphate buffer for 2 h at room temperature. The sample was then washed two times with 0.1  M cacodylate buffer and post-fixed with 1.0% osmium tetroxide for 1 h at room temperature. After this second fixation step, the sample was washed three more times with 0.1 M cacodylate buffer and immersed in 0.1% tannic acid for 20 min. It was then washed again with a cacodylate buffer and dehydrated through a graded ethanol series (35% to 100%). Critical point drying with CO_₂_ was subsequently performed, followed by mounting on stubs and sputter-coating with a 5-nm gold layer. Imaging was carried out using a FEI Quanta 200 scanning electron at the Microscopy Center of UFMG.

### Viral genome extraction, sequencing, assembly, and annotation

DNA extraction from the viral isolate was performed using the QIAamp MinElute Virus Spin Kit (Qiagen, Germany). The extracted viral DNA was sequenced using the Illumina MiSeq platform with a single-end library, prepared using the Illumina DNA Prep kit (Illumina Inc., San Diego, CA, USA), and IonTorrent platform, with libraries prepared using Ion Shear, Ion Plus Fragment Library, and E-gel 2% kits (ThermoFisher Scientific, USA). Quality control of the obtained reads was conducted using FastQC, and low-quality bases were removed using Trimmomatic version 0.36.3 (20). *De novo* genome assembly was carried out with SPAdes version 3.12, using default parameters (21, 22). The assembled scaffolds corresponding to the viral isolate were selected and scaffolded based on the reference genome of the chlorovirus CviKI using the MeDuSa online platform v1.6 (accessed 8 April 2025) (23).

Open reading frame (ORF) prediction was performed using GeneMarkS (accessed 4 May 2025) (24), considering only sequences with more than 40 amino acids. Predicted sequences were annotated using BLASTp with nr database (accessed 11 May 2025) (25), HHpred v2.08 with PDB database (accessed 11 May 2025) (26), and InterProScan v104.0 with all applications (27). Following annotation, predicted proteins were categorized based on the functional classification of NCLDV orthologous groups of genes (NCVOGs) (2). For proteins not present in the reference database, manual annotation was performed using the UniProt platform (accessed 11 May 2025) (28). To allow for a proper comparison with the PBCV-1 proteome, we used the same strategy for *de novo* gene prediction and viral genome annotation, using the Reference Sequence (GenBank accession number: NC_000852.5). Predicted proteins of the new virus annotated as ORFans or hypothetical and larger than 200 amino acids were selected to be used in a molecular modeling pipeline to identify possible structural homologies. We included only predicted proteins of larger size to increase our chances of obtaining a reliable result and finding homologs in databases. Each sequence was used as a query in Swiss-Model (29), Modeler (30), and AlphaFold2 (31) (via ColabFold). For targets modeled in AlphaFold2, the FoldSeek server (32) was used to identify homologous structures. Only models with acceptable parameters for each program were accepted. The models were visualized and edited in PyMol 3.1.5.1 (33). Sequences identified as enzymes were selected for binding site identification using FTMove (34).

### Phylogenetic analyses

To perform the phylogenetic analysis of the viral isolate, a maximum likelihood reconstruction was conducted using IQ-TREE v2.3.5 with 1,000 bootstrap replicates (35). The data set was assembled by performing BLASTp searches against the NCBI database, using the DNA polymerase sequence of the isolate along with sequences from other chloroviruses and outgroup organisms. Multiple sequence alignment was performed using the MUSCLE algorithm implemented in the MEGA 11 software (36, 37). LG + I + R4, the best-fit substitution model, was determined using ModelFinder implemented in IQ-TREE (38). The resulting phylogenetic tree was visualized and edited using iTOL v6 (39).

### Comparative genomic analyses

The Dynamic Genomic Alignment server (DiGAlign) (40) version 2.0 was used for analyzing the alignment of the genomes of the *C. vanettense* species and genomic synteny. For this analysis, the genomes of some isolates of this species deposited in Genbank were used as the input (accessed 24 August 2025), in conjunction with the new virus genome. Average nucleotide identity (ANI) was calculated using FastANI v1.3 (41) implemented at the European Galaxy server (usegalaxy.eu). For the calculation of average amino acid identity (AAI), the EzAAI v1.2.4 (42) software was employed. The resulting data were organized into a mixed similarity matrix (ANI × AAI), which was used as input for the construction of a heatmap using in-house scripts written with the numpy, seaborn, and matplotlib packages for Python, allowing similarity comparison of the new isolate in relation to the other 21 isolates of the species *Chlorovirus vanettense*.

### Pan-genome construction

The pan-genome was constructed using OrthoFinder v3.0.1b1, which was employed to cluster the previously predicted CDS into orthologous groups (COGs), with an MCL inflation parameter fixed at 4 (43). The CDS predictions of the 22 isolates of the *C. vanettense* species were used as the input in this analysis. Each prediction was incorporated into the analyses in sequence, considering the order of the isolates in the ANI matrix. A pan-genome considering all isolates of *Alphachlorovirus* subgenus was built using OrthoFinder with the same parameters indicated above. For COG-sharing visualization, we built a bipartite network using Gephi v0.10.1 (44), applying the force-based algorithm ForceAtlas2 with further minimal manual organization of the nodes to reveal the singletons.

## RESULTS

### Isolation and characterization of the first giant virus of microalgae in Brazil

**Fig 1:**
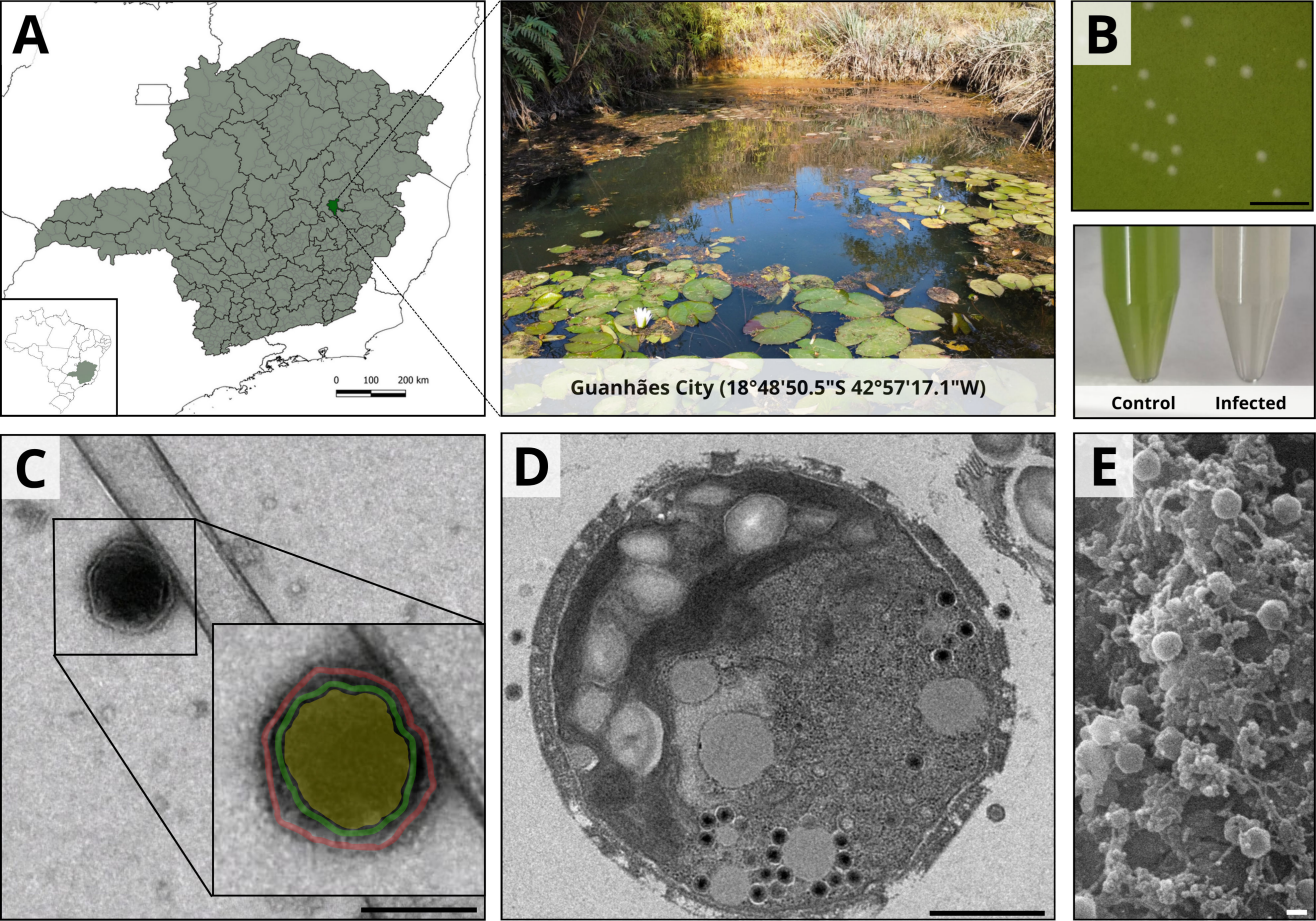
Isolation and morphological characterization of *Chlorella* virus isolate BR-MG-N01. (**A**) Isolation site of the Brazilian *Chlorella*-infecting virus. QGIS software was used to create the Minas Gerais map. The photo shows the lake where the sample was collected and geographic coordinates are indicated. (**B**) BR-MG-N01 plaque morphology and effect in infected culture; the scale bar indicates 1 cm. (**C**) Negative staining electron microscopy of BR-MG-N01, revealing a particle of approximately 160 nm in diameter. Magnification: in red—viral capsid, in green—inner membrane, and in yellow—viral genome. The scale bar indicates 200 nm; the image was digitally colored. (**D**) TEM image of *Chlorella variabilis* NC64A infected with BR-MG-N01. A high number of viral particles forming in the cytoplasm of the cell can be observed, along with the disruption of its cell wall and the release of viral particles; the scale bar indicates 1 µm. (**E**) SEM image of BR-MG-N01 particles. Cell debris with a high number of attached icosahedral particles can be observed; the scale bar indicates 100 nm.

Among the assays performed, we observed cytopathic effects in a *Chlorella variabilis* NC64A plate after enrichment with a lake water sample from Guanhães City (Fig. 1A). The resulting isolate was the first *Chlorella*-infecting virus identified in Brazil utilizing this algal species and was designated BR-MG-N01, referring to the country (BR – Brazil), state (MG – Minas Gerais), and host cell strain of isolation (N – NC64A). The virus formed lysis plaques approximately 2 mm in diameter in solid medium assays 48 hpi, and in liquid medium, it causes complete clearing of the algal culture within 7 days post-infection (Fig. 1B).

Following production and purification, we sent the BR-MG-N01 isolate to the Microscopy Center of UFMG for transmission and scanning electron microscopy analyses to assess the morphology and size of the viral particles. The micrographs revealed a high number of electron-dense icosahedral particles, approximately 160 nm in diameter. In TEM analysis at higher magnification, a clear distinction could be seen between the outer viral capsid and the internal lipid membrane, a structural hallmark of chloroviruses (1). A region of high electron density was observed within the viral particle, representing the virus DNA (Fig. 1C).

After 24 hpi, electron-dense particles were detected within the cytoplasm of infected cells, organized into viral factories (Fig. 1D), regions commonly observed in chlorovirus-infected cells (1). In addition, certain host organelles exhibited structural alterations following infection. Thylakoids became disorganized and separated, and the cytoplasm of infected cells contained lipid inclusions and starch granules. A large number of viral particles were also observed using scanning electron microscopy analysis (Fig. 1E).

### Gene repertoire and evolution of chlorovirus BR-MG-N01

**Fig 2:**
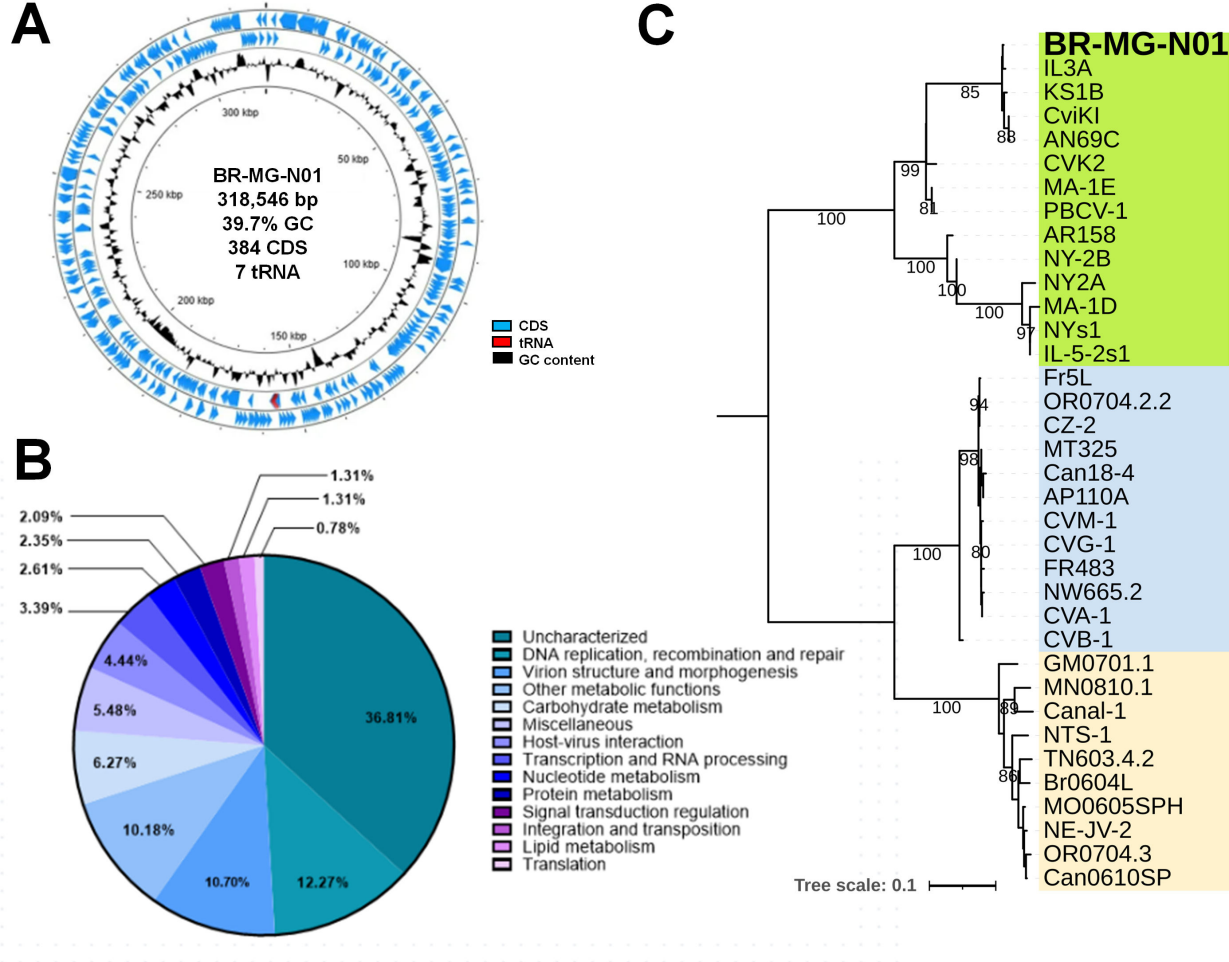
Genomic features and phylogeny of chlorovirus BR-MG-N01. (**A**) Genomic map of BR-MG-N01. CDSs are shown in blue, tRNAs in red, and GC content variation in black. (**B**) Gene function distribution of the BR-MG-N01 isolate. The pie chart shows the percentage distribution of the viral genome across 14 functional metabolic categories described for nucleocytovirus orthologous groups of genes. (**C**) Phylogenetic reconstruction based on DNA polymerase B family gene. The BR-MG-N01 isolate is highlighted in bold. Green represents the Alphaclorovirus, blue the Betachlorovirus, and yellow the Gammachlorovirus subgenera. The tree was rooted using betachlorovirus and gammachlorovirus as outgroups. The scale bar represents the amino acid substitution rate.

Following viral DNA extraction and sequencing, a total of 1,090,424 high-quality reads were generated. After *de novo* assembly using SPAdes, seven scaffolds were obtained for the viral genome. A reference-guided assembly of the seven scaffolds using the genomes of chloroviruses CviKI and MA-1E (best hits using Blastn search) resulted in a single scaffold of 318,547 bp with a GC content of 39.7%, 384 coding sequences (CDSs), and 7 tRNAs *in tandem* in the middle of the genome, confirming this new isolate as chlorovirus, that is, a new member of the genus *Chlorovirus* (Fig. 2A). Notably, 36.8% of the viral genome consisted of genes with no known function, including two unique genes (i.e., ORFans) (Fig. 2B). Prominent functional categories within the genome included those related to DNA replication, repair, and recombination (12.27%), as well as carbohydrate metabolism (6.27%), a pattern consistent with known chloroviruses (1, 45). Among the carbohydrate metabolism genes, the most frequently identified were those related to hydrolytic enzymes, such as alginate lyases and chitinases, which are likely involved in the viral infection cycle (46, 47). Among the known genes, the BR-MG-N01 isolate showed a higher number of genes in several functional categories compared with PBCV-1. These include DNA replication, repair, and recombination (47 vs 35 genes); virus–host interaction (18 vs 9); integration and transposition (5 vs 1); lipid metabolism (5 vs 3); nucleotide metabolism (10 vs 6); and RNA transcription and processing (13 vs 11) (Fig. 2B; also see Fig. S1 and Table S1 at https://doi.org/10.6084/m9.figshare.31410741). Within the DNA metabolism category, 17 genes encoding GIY-YIG domain endonucleases were present. About 10% of the BR-MG-N01 gene content was related to virion structure. We identified homologs for 23 capsid genes identified in PBCV-1 (48), suggesting a similar structure composition of the Brazilian isolate. Genome annotation of BR-MG-N01 revealed the presence of genes related to antibiotic resistance (chloramphenicol acetyltransferase), oxidative stress response (Cu/Zn superoxide dismutase), and transcription processes (mRNA capping enzyme and RNAse).

From sequences with no known function, i.e., ORFans, hypothetical proteins, and proteins containing domains of unknown functions (DUFs), we conducted a series of modeling procedures using different platforms, attempting to identify any function to those genes. Among those used, only AlphaFold2-generated models with reasonably acceptable parameters for only 2 of 34 analyzed genes having more than 200 aa (see Table S2 at https://doi.org/10.6084/m9.figshare.31410741). According to FoldSeek, an endonuclease from chlorovirus CVG-1 (code BFVD MH1GT3) showed homology with the two generated models, which suggests the possibility of assigning this function to the related genes. To add more reliability to the obtained result, we used SwissModel to align the primary sequences of the genes in question to the MH1GT3 template, using the server’s “user template” option. The obtained Global Model Quality Estimation (GMQE) scores were 0.41 for gene 71 (46% identity and 98% coverage) and 0.42 for gene 379 (46% identity and 98% coverage). Therefore, the template identified in this study was also generated by AlphaFold2, which requires caution when inferring homology and function.

Phylogenetic analysis of the DNA polymerase gene (*a185r* in PBCV-1) using 37 sequences (including beta and gammachloroviruses outgroup) placed the BR-MG-N01 isolate within the *Alphachlorovirus* subgenus, closely related to *Chlorovirus vanettense* (same species as PBCV-1) (Fig. 2C). The topology was consistent with established clades, and high bootstrap support (>90%) corroborated the assignment.

### Comparative genomics and pan-genome of *Chlorovirus vanettense*

**Fig 3:**
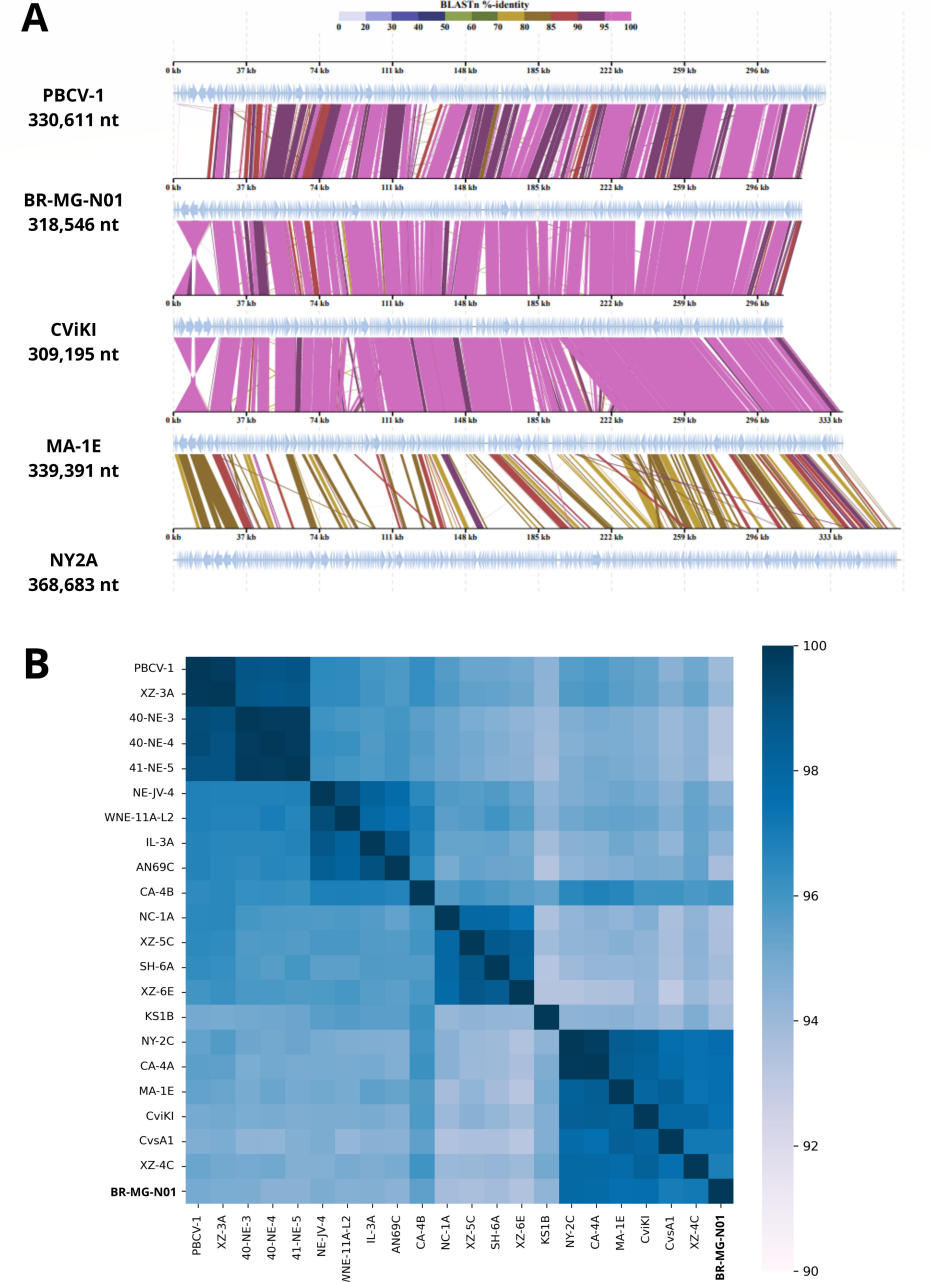
Genome synteny and identity between alphachlorovirus isolates. (**A**) Genome synteny analysis using four isolates of the species *Chlorovirus vanettense* and one isolate of *Chlorovirus americanus*. Deletions and inversions are observed among the isolates. Colors represent the percentage of similarity across the genome. (**B**) Similarity matrix of mean ANI and AAI among isolates of the species *Chlorovirus vanettense*. Color range represents the degree of similarity among the isolates.

The synteny analysis showed genome identity conservation of the isolate compared with the other two analyzed isolates of the species *Chlorovirus vanettense* (Fig. 3A). A notable difference was observed at the beginning of the BR-MG-N01 genome, which contains a region absent in the PBCV-1 genome and an inverted region compared with chlorovirus CViKI. In addition, genome-wide identity fluctuates more when compared with PBCV-1. These differences may be related to the distinct biological features of BR-MG-N01 compared with the model isolate while still showing the greatest similarity with other isolates of the species. A notable difference can be observed when comparing the genomic synteny identity of these isolates with chlorovirus NY2A, a member of the species *Chlorovirus americanus*. The similarity matrix indicated that the isolate has high genomic proximity to isolates of the species *Chlorovirus vanettense*, particularly NY-2C, CA-4A, MA-1E, CViKI, CvsA1, and XZ-4C, with which BR-MG-N01 shares more than 98% identity, thus confirming it as a member of the species *Chlorovirus vanettense* (49) (Fig. 3B). Interestingly, even within the same species, four blocks of very high similarity (>98%) were observed among isolates, some of which were obtained from samples collected in geographically distant locations, such as Minas Gerais (BR-MG-N01), California (CA-4A), and New York (NY-2C).

**Fig 4:**
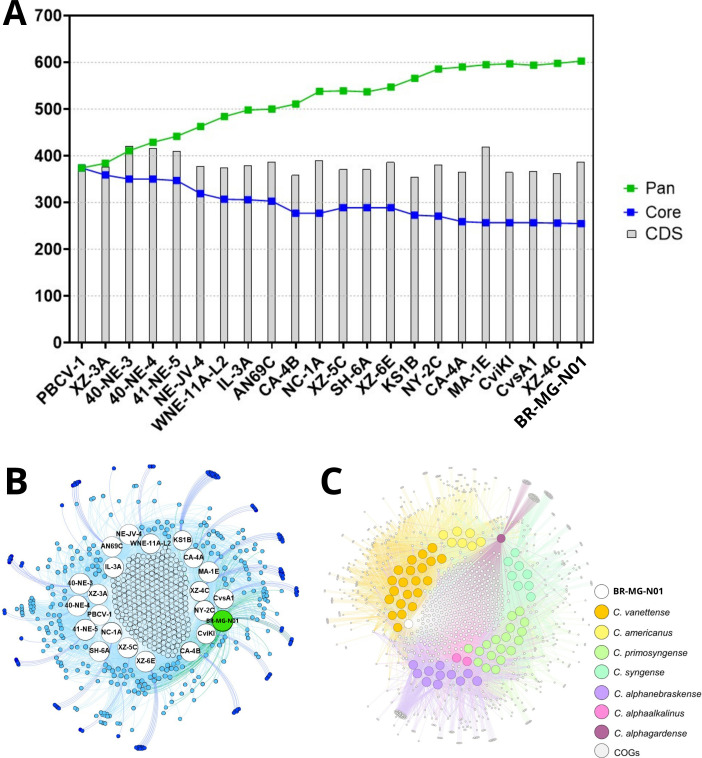
Pan-genome and COG-sharing pattern in *C. vanettense*. (**A**) Pan-genome evolution of *Chlorovirus vanettense*. The evolution of the pan-genome is shown in green, and the evolution of the core genome in blue. Gray bars indicate the number of CDSs in each isolate. (**B**) Network graph representing the distribution pattern of COGs in *C. vanettense*. White circles represent the isolates, light circles in the center represent the core genome, light blue circles represent the satellite genome, and dark blue circles at the edges represent the singletons. (**C**) Network graph representing the distribution pattern of COGs between alphachloroviruses species. Larger circles represent the isolates of each chlorovirus species. Each color indicates a different species. The white circle represents the BR-MG-N01 isolate. Smaller circles indicate the COGs.

We evaluated the evolution of the *Chlorovirus vanettense* pan-genome and investigated whether isolating and characterizing a virus from a region with no prior knowledge of chloroviruses would yield any genomic novelty. Considering the 22 isolates of the species, a total of 8,415 genes were grouped into 603 orthologous groups (COGs), of which 255 constitute the core genome of *Chlorovirus vanettense* (Fig. 4A and B). Seventy-eight COGs were singletons within the species, of which five were associated with BR-MG-N01, revealing that the new isolate contributes to the expansion of the viral species' pan-genome. All isolates have at least one singleton, indicating that each isolate has unique genes that contribute to the expansion of the pan-genome (Fig. 4B). Considering all alphachloroviruses (*n* = 69), a total of 27,423 genes were grouped into 979 orthologous groups, of which 150 are singletons. A total of 179 COGs is shared among all isolates, constituting the core genome of alphachloroviruses (Fig. 4C). The pattern of COG-sharing among isolates reinforces the taxonomic classification of alphachloroviruses into seven species, where we observed that isolates sharing a greater number of genes are closer together in the network graph. Chlorovirus BR-MG-N01 shared the most COGs with isolates belonging to the species *Chlorovirus vanettense*, as expected. Interestingly, even considering all 69 isolates, we observed that the Brazilian isolate had two singletons, indicating that, even with a large amount of genomic data, a new isolate can still contribute genomic novelty to the group under investigation.

### BR-MG-N01 exhibits biological differences compared with the type chlorovirus PBCV-1

**Fig 5:**
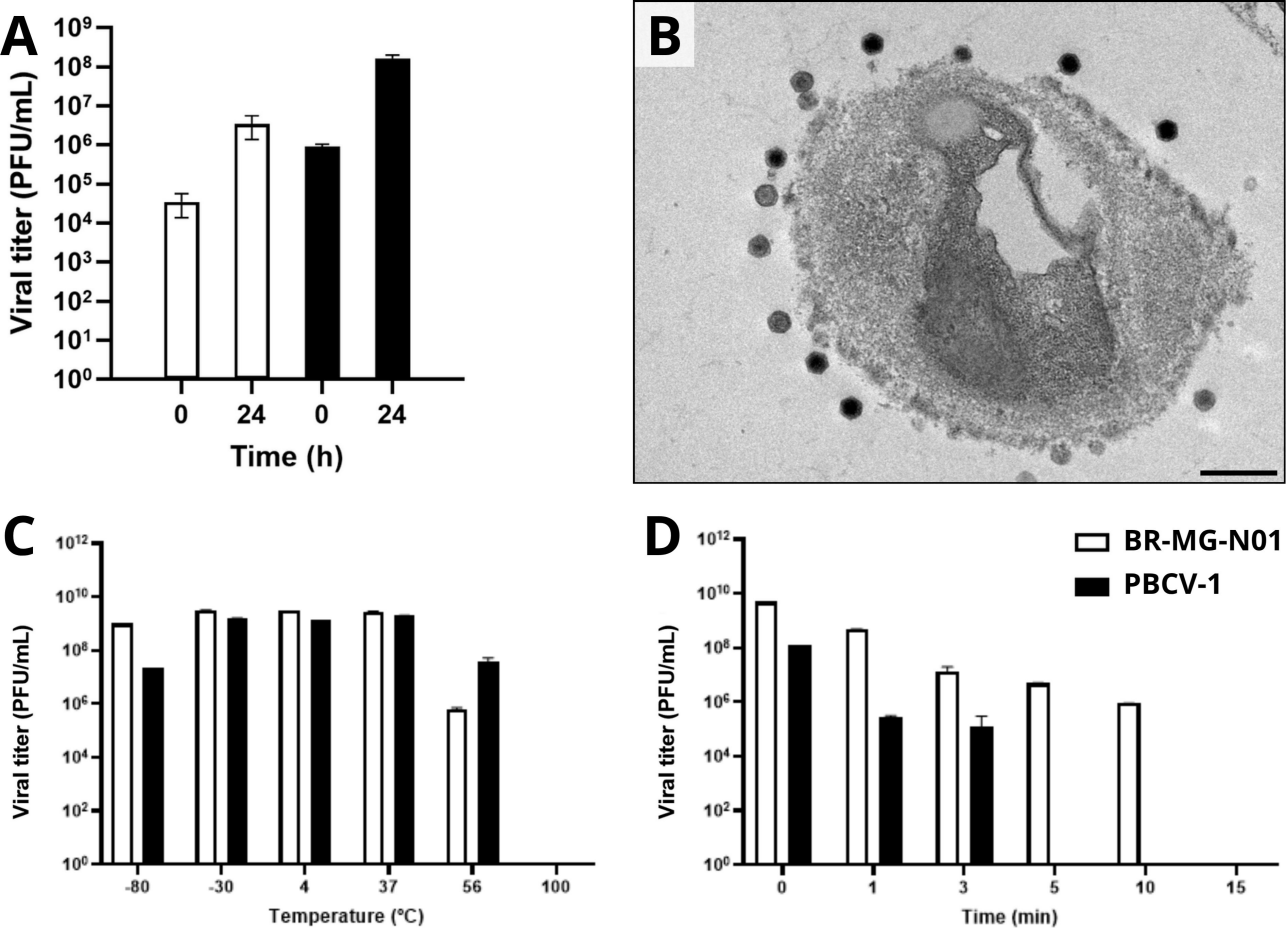
Biological characteristics of BR-MG-N01. (**A**) Viral yield of BR-MG-N01 and PBCV-1, 24 hpi using an MOI of 10 PFU per cell. (**B**) Transmission electron microscopy of a *Chlorella variabilis* NC64A cell infected with BR-MG-N01 at 24 hpi. The image shows a lysed cell releasing several viral particles of approximately 160 nm in diameter; the scale bar indicates 500 nm. (**C**) Stability assay of the BR-MG-N01 isolate at different temperatures. (**D**) Stability assay of the BR-MG-N01 isolate under UV radiation exposure. Graphs correspond to mean values obtained from two independent biological replicates. Error bars indicate standard deviation.

To assess the replication profile of BR-MG-N01, infections were carried out at a MOI = 10. The new isolate showed a 2-log_10_ increase after 24 h (Fig. 5A). When compared with PBCV-1, the replication kinetics of BR-MG-N01 appeared very similar to those of the model alphachlorovirus, which exhibited a 3-log_10_ growth under similar conditions after 24 h. TEM analysis of infected cells after 24 h evidenced cell lysis with multiple electron-dense BR-MG-N01 particles outside the cell (Fig. 5B). Cell debris and dispersed viral particles, resulting from previous infections, are also visible in the sections.

To assess the stability of the BR-MG-N01 isolate at different temperatures, infections were performed following exposure of viral stocks to −80°C, −30°C, 0°C, 37°C, 56°C, and 100°C for 10 min. The isolate remained viable across the temperature range of −80°C to 56°C. At −80°C, the viral titer was reduced by one-third compared with the initial value, decreasing to 1 × 10⁹ PFU/mL. Using treatments from −30°C to 37°C, the titer remained stable at 3 × 10⁹ PFU/mL. However, treatment with 56°C, the titer dropped significantly to 8 × 10⁵ PFU/mL. A similar profile was observed for PBCV-1, but it appeared to be slightly more tolerant at 56°C. Both viruses were completely inactivated at 100°C (Fig. 5C). When exposed to ultraviolet radiation, chlorovirus BR-MG-N01 showed a sharp decrease in viral titer after just 3 min of exposure. Starting from an initial titer of 3 × 10⁹ PFU/mL, the virus was reduced to 4.8 × 10⁷ PFU/mL after 1 min, 1.8 × 10⁶ PFU/mL after 3 min, 4.9 × 10⁵ PFU/mL after 5 min, and 8.9 × 10⁴ PFU/mL after 10 min. The virus was inactivated after 15 min of UV exposure (Fig. 5D). Curiously, PBCV-1 showed even less resistance to UV radiation, being completely inactivated after only five min of exposure.

## DISCUSSION

The knowledge about chloroviruses has grown considerably in recent years, with the discovery of dozens of new isolates from different regions of the world and the advancement of studies and tools for genomic analyses of these viruses. Despite these advances, a substantial gap remains regarding the biology, evolution, and genomics of chloroviruses, particularly in regions outside North America. Here, we report the isolation and characterization of a Brazilian chlorovirus isolate, designated BR-MG-N01, which was able to form lysis plaques in solid medium and completely clear cultures of *Chlorella variabilis* in liquid medium. The isolate belongs to the *Alphachlorovirus* subgenus, showing high similarity (>94%) with isolates of the species *Chlorovirus vanettense*, the same species as the model isolate PBCV-1. The virus exhibited an icosahedral capsid of approximately 160 nm, slightly smaller than that of PBCV-1, with structural features consistent with those of other chloroviruses (50).

Chlorovirus BR-MG-N01 demonstrated thermal resistance at 56°C, similar to thermophilic organisms, and remained viable at temperatures below 0°C. This feature is particularly noteworthy, as it may suggest that these viruses could inhabit colder environments, such as polar regions, infecting native microalgae and displaying significant biological and genomic differences, likely due to geographic isolation and divergent environmental conditions. It is important to mention that we submitted the virus to different temperatures for a short period of time, and exposure for longer periods could affect viral stability. Conversely, BR-MG-N01 showed little resistance to UV radiation under the conditions tested, although this resistance was higher than that observed for the model PBCV-1. This resistance may represent an important biological and evolutionary strategy for these viruses, since the environments inhabited by their hosts are exposed to sunlight for extended periods. Increased resistance could provide greater stability to viral particles outside algal cells, maintaining active populations across diverse environments and for longer periods, directly impacting their ecological roles in controlling host population and fueling the microbial food web.

Chlorovirus BR-MG-N01 displayed a replication profile very similar to that of PBCV-1 (51). TEM analyses of asynchronous infection revealed cells at different stages of viral replication. Previous studies indicate that alginate lyases act in conjunction with other enzymes at the onset of infection, degrading the microalgal cell wall so that the virus can fuse its inner membrane with the host membrane, thereby releasing its genome into the cytoplasm of the infected cell (52, 53). As the infection progressed, an increase in the cytoplasmic area of the host cell was observed, accompanied by a reduction in chloroplast area. Interestingly, in infected cells where viral factories were visible, large starch granules were found in the cytoplasm of the microalgae, surrounded by assembling viral particles. In plants, a suppression of starch accumulation is generally observed during the early stages of viral infection (54). In chloroviruses, however, starch accumulation may result from viral manipulation of the host microalga’s metabolism. The reduction in chloroplast integrity and the possible inhibition of enzymes involved in photosynthesis and sugar synthesis may prevent normal starch metabolism, leading to its retention at higher concentrations in the cytoplasm of infected cells. Further studies are required to elucidate this observation.

The new isolate has a genome structure and identity percentage similar to those of other alphachloroviruses. When compared with the closest genomically related isolates, NY-2C and CA-4A, which share more than 97.5% of genome identity, the Brazilian isolate has a larger genome (BR-MG-N01: 318 kpb, 384 CDSs) than the California isolate (CA-4A: 313 kbp, 367 CDSs) and a smaller genome than the New York isolate (NY-2C: 323 kbp, 373 CDSs). PBCV-1 also has a larger genome (330 kbp) but encodes fewer CDSs (*n* = 377). Some alphachlorovirus isolates share >99% genome identity (e.g., NY-2C and CA-4A, and PBCV-1 and XZ-3A). Previous studies have already shown that within betachloroviruses, genetic variants or genomovars can be found (14). Despite their extremely high genetic similarity, some of these variants exhibited major biological differences, particularly in the plaque morphology observed. Within gammachloroviruses, a high similarity is observed between isolates from the Southern Hemisphere (Brazil and Chile) and those obtained in the Northern Hemisphere, whereas isolates from the same locality (Nebraska, USA) show high genetic divergence (15). Overall, chlorovirus diversity does not appear constrained by geography, and genomic analyses are essential for defining species. Broader sampling, especially in underexplored regions, is needed to uncover the factors shaping their diversity. In addition, the biological differences found between BR-MG-N01 and PBCV-1 highlights that although the genome information is essential for knowledge about their evolution, it might not be enough to understand the biological diversity of these viruses in nature.

Considering the genetic content of chlorovirus BR-MG-N01, genes involved in DNA replication, repair and recombination were the most abundant, consisting of more than 12% of the virus gene pool. Compared with the model PBCV-1, the main difference observed was the high presence of GIY-YIG domain endonucleases in BR-MG-N01, which are less prevalent in PBCV-1. These proteins are distributed across diverse organisms, performing multiple functions (55). In bacteria, GIY-YIG domain endonucleases have been identified that cleave phosphodiester bonds in damaged DNA fragments (56). The protein is able to recognize a specific DNA target using minimal elaboration of the core fold (57), which may be useful for repairing viral progeny DNA during replication in the host cell. If confirmed, this difference could imply a greater capacity for repairing genetic material damage in the Brazilian isolate, which may help explain the higher stability of BR-MG-N01 at low temperatures and under UV exposure, as well as a lower capacity to produce infectious particles compared with PBCV-1. Gene annotation also revealed nine additional HNH endonucleases, further highlighting the abundance of these genes in the BR-MG-N01 genome. It is possible that the isolate employs these endonucleases to evade host defense mechanisms, as well as to cleave the DNA of competing viruses, thereby providing a selective advantage (58). Following annotation through sequence alignment analyses against databases, a structural modeling analysis of ORFans further suggested the possible presence of two additional putative endonucleases in the BR-MG-N01 genome.

The annotation revealed the presence of a chloramphenicol acetyltransferase gene, which is associated with antibiotic resistance in bacteria. This finding is relevant in light of recent studies showing that antibiotic resistance genes (ARGs) can occur in giant viruses from different families, such as *Mimiviridae*, *Marseilleviridae*, and *Phycodnaviridae* (59). These genes may have been acquired through horizontal gene transfer from microorganisms associated with the host, potentially playing additional roles in the viral life cycle beyond antibiotic resistance itself. This observation reinforces the idea that chloroviruses, although not classical pathogenic agents, share with other nucleocytoplasmic large DNA viruses (NCLDVs) an underestimated genomic complexity, and that their genomes may carry resistance-related genes with possible ecological and evolutionary implications. A gene encoding Cu/Zn superoxide dismutase linked to the neutralization of reactive oxygen species (ROS) was also identified. Among large DNA viruses, homologs of this gene are also found in poxviruses, baculoviruses, and mimiviruses, as well as in some chloroviruses, such as PBCV-1 (60). The presence of this gene in chloroviruses appears to be related to a faster replication cycle and a larger burst size. The BR-MG-N01 isolate may use this gene to subvert host ROS, acting as a viral resistance mechanism against cellular defenses during infection. In addition, genes related to transcription were identified, such as mRNA capping enzyme and RNAse, indicating that BR-MG-N01, like other chloroviruses, has genes involved in these processes, although they lack an RNA polymerase.

Considering the GMQE threshold of >0.6, our results must be interpreted with caution. This raises the question of whether we are currently facing a gap in chlorovirus gene annotations: can functions still be reliably assigned to genes of unknown function based solely on predictive data? Our analyses indicate that this is not the case. Possibly, this dark matter of data is composed of completely unknown genes, with no homologs within the currently discovered viral and cellular diversity. In this sense, we recognize the need for efforts involving transcriptomic and proteomic studies of chloroviruses, beyond PBCV-1, as well as other *in vitro* assays involving the expression and characterization of these genes. A prime example is the work of Shao et al. (48), which discovered proteins related to the capsid of this viral group, making it possible to correlate them with sequences that had no defined function until then. These data were used in the refined annotations made by Carvalho et al. (2) and Henriques et al. (15), which resulted in percentages related to the NCVOG “uncharacterized function” of 45.37% and 40%, respectively, showing an improvement compared with the 47% described by Rodrigues et al. (3). Placing the work described here within this historical perspective, the success of this refinement in discovery techniques is evident, as a virus with only 36% unknown proteins has now been described, using algorithms benchmarked in previous studies.

Finally, the isolation and characterization of a Brazilian chlorovirus raises important questions about the viral distribution in unexplored geographic regions and interaction with their hosts in different environments. Chlorovirus BR-MG-N01 was isolated using the microalga *Chlorella variabilis* NC64A as lab host. This algal species was originally collected in North America (61), and there are no reports in the literature of its occurrence in Brazilian waters. It is possible that species phylogenetically or structurally related to the one used in this study exists in the environment where the sample was collected (Guanhães City, Minas Gerais) and, in this case, serving as the natural host of this chlorovirus. Another hypothesis is the presence of *Chlorella variabilis* in waters of Minas Gerais, which would represent a novel finding in terms of our biodiversity knowledge of Brazilian aquatic environments. In the future, the characterization of the water sample in its biotic and abiotic aspects along with the description of the surrounding areas might be important to better understand the tropism of environmental viruses. Further analyses may be conducted to explore these biomes, helping to fill existing gaps in our knowledge of microorganisms and their enigmatic viruses.

## Data Availability

The data sets generated and/or analyzed during the current study are available in the GenBank repository. The raw sequencing data and genome of Chlorovirus BR-MG-N01 have been deposited in the BioProject PRJNA1347844, SRA SRR35846387, and under accession number PX673726.1. The supplemental materials are available at FigShare: 10.6084/m9.figshare.31410741.
